# Development, Validation, and Use of ^1^H-NMR Spectroscopy for Evaluating the Quality of Acerola-Based Food Supplements and Quantifying Ascorbic Acid

**DOI:** 10.3390/molecules27175614

**Published:** 2022-08-31

**Authors:** Asma Bourafai-Aziez, Daniel Jacob, Gwladys Charpentier, Emmanuel Cassin, Guillaume Rousselot, Annick Moing, Catherine Deborde

**Affiliations:** 1 EVEAR EXTRACTION, 48 Route de Gennes, LD Félines, CEDEX 4, F-49320 Coutures, France; 2 INRAE, Univ. Bordeaux, Fruit Biology and Pathology, UMR1332, 71 Avenue E. Bourlaux, F-33140 Villenave d’Ornon, France; 3 Bordeaux Metabolome, MetaboHUB, PHENOME-EMPHASIS, 71 Avenue E. Bourlaux, F-33140 Villenave d’Ornon, France

**Keywords:** acerola, metabolomic profiling, ascorbic acid, vitamin C, qNMR, food supplement

## Abstract

Acerola (*Malpighia emarginata* D.C.) is an exotic fruit with high agro-industrial potential due to its high content of ascorbic acid (AA), phenolic compounds, and carotenoid pigments. Acerola fruit is processed into concentrated juice or powder to be incorporated into food supplements. The ascorbic acid content of concentrated juice or powders must be controlled and well assessed. Therefore, the development of optimal methods and procedures for the rapid and accurate determination of the ascorbic acid content in juice concentrate and juice powder remains of considerable commercial interest. NMR spectroscopy is currently a powerful spectroscopic tool for the qualitative and quantitative analysis of molecules of all types and sizes. Firstly, this article presents the NMR-based metabolomic profiling of acerola juice and concentrate powder to describe and compare their composition. Thirty-six metabolites were identified. The AA over choline ratio and the NMR metabolomic profiles could be used for authentication in the future. Secondly, a rapid (8 min), reliable, and non-destructive method for the quantification of ascorbic acid by 1D ^1^H-NMR spectroscopy was developed and validated. The LOD and LOQ were 0.05 and 0.15 mg/mL, respectively. These two approaches could be combined to better characterize ingredients derived from acerola and incorporated into food supplements.

## 1. Introduction

Acerola (*Malpighia emarginata* D.C.) is a small tree that grows in dry deciduous forests. It is native to central and northern South America and has been cultivated in large areas of Brazil [1,2]. Its red fruit, which resembles the European cherry, contains about 80% juice and a large amount of vitamin C (500–4500 mg/100 g) [3]. It is also rich in other vitamins and nutrients such as thiamin; riboflavin; niacin; carotenes; phenolics; proteins; and mineral salts, mainly iron, calcium, and phosphorus [4]. Its vitamin C content depends on the season, climate, growing site, and, in particular, the ripening stage: the riper the fruit, the lower its vitamin content. Unripe fruit can contain up to 4.5% vitamins, 90 times more than peeled oranges [5,6]. 

Since the human body does not synthesize ascorbic acid (AA), it must be obtained via external sources [7]. This is why vitamin C is considered one of the most significant vitamins, taking part in several biological functions, such as enhancing collagen formation [8,9,10]. Therefore, acerola high content in vitamin C content makes it one of the best sources of natural antioxidants, helping to prevent many diseases and delay aging [10,11].

As a result, many food supplements are manufactured from acerola juice. Their quality depends on the quantity of key active components and the absence of undesirable materials such as adulterants and residual solvents. Claims of benefits depend on specific molecules in the extracts, which must therefore be identified and quantified with great precision. 

In acerola or acerola-derived samples, the quantification of vitamin C, mostly composed of AA, is generally carried out by destructive targeted analytical methods: either liquid chromatography (HPLC), coupled with several detection methods such as ultraviolet or electrochemical detection [12,13], or spectrofluorometric methods [14,15]. These methods each have advantages and drawbacks for the targeted quantification of ascorbic acid [15]. In 2009, a time-consuming quantitative analysis method for ascorbic acid by ^13^C-NMR was proposed. This method requires a large amount (500 mL) of acerola juice to be processed in order to purify the AA by preparative HPLC before ^13^C NMR analysis [5]; therefore, it is not suitable for a large series of samples. However, due to its sensitivity to temperature, pH, oxygen concentration, and light intensity changes, vitamin C is considered unstable [16], which presents a fundamental limitation for these analytical methods. Moreover, these methods do not reveal the whole composition of the acerola-derived ingredient or food supplement and therefore do not allow the detection of potential fraud. Thus, the development of optimal approaches and procedures for rapidly and accurately quantifying the AA content in acerola juice concentrate and spray powder from juice concentrate is of enormous scientific and commercial interest. 

Nuclear magnetic resonance spectroscopy (NMR) has been used in food quality control after the extraction of target compounds (e.g., ethanol by SNIF-NMR for chaptalization detection [17], vanillin for authentication and origin determination [18], and ascorbic acid for authentication and origin determination [5]) or to analyze mixtures such as milk, fruit juice, wine, and oil [19]. NMR-based metabolomics is a commonly used approach for the identification and quantification of metabolites or compounds in food extracts [20,21]. NMR spectroscopy has been widely used to characterize the composition of plant extracts as a qualitative and quantitative tool. Metabolome analyses based on NMR spectroscopy allow the efficient identification and quantification of new and known major metabolites [22,23,24,25,26]. Recently, the metabolomic profiling of acerola fruits in different ripening stages was determined using proton NMR (^1^H-NMR) [6]. To our knowledge, no NMR metabolomic studies have been performed to verify the authenticity and content of ascorbic acid in concentrated juices and acerola-based formulations. 

In this context, the first objective of this work was to establish, by NMR, the metabolomic profiles of acerola juice concentrate, spray-dried acerola juice powders, and acerola food supplement formulations in order to describe and compare the detailed composition and origin of the analyzed samples. The resulting metabolomic profiles could be used for future authenticity control. The second objective was to develop and validate a rapid, reliable, and non-destructive method for the quantification of ascorbic acid by mono-dimensional (1D) ^1^H-NMR spectroscopy.

## 2. Results and Discussion

### 2.1. Qualitative Analysis 

#### 2.1.1. NMR-Based Metabolomic Profiling of Acerola Juice Concentrate

**Figure 1:**
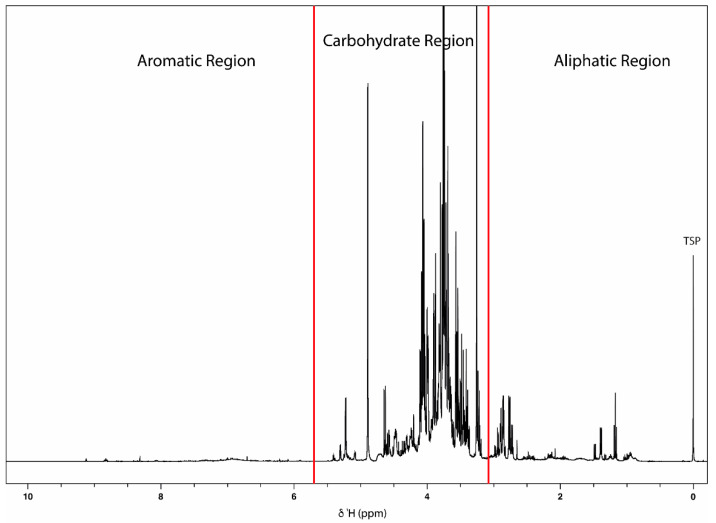
Representative 1D ^1^H-NMR spectrum of a typical aqueous sample of acerola juice concentrate (400 MHz, 300 K, D_2_O, pH 3.35, 20 mM TSP).

The present study used acerola juice concentrate for metabolic profiling via an untargeted metabolomic approach. Figure 1 shows a representative 1D ^1^H-NMR spectrum of acerola juice concentrate. The spectrum contains well-resolved and intense signals for low-molecular-weight metabolites in the aliphatic region between 0.5 and 3.0 ppm and interleaved sugar signals between 3.0 and 5.5 ppm. In the aromatic region (5.5 to 9.5 ppm), low-intensity signals are detected.

The 1D ^1^H-NMR spectrum resonances were first assigned according to corresponding literature data [6,27] as well as public (HMDB [28], BMRB [29]) and in-house NMR databases of pure compounds and with spiking experiments. Then, considering the signal overlap observed in the spectrum, a set of 2D NMR experiments (JRES, TOCSY, ^1^H-^13^C HSQC, and ^1^H-^13^C HMBC) were recorded. Table 1 lists the chemical shift data for more than 30 metabolites that were identified, with their level of identification according to Sumner et al. [30].

**Figure 2:**
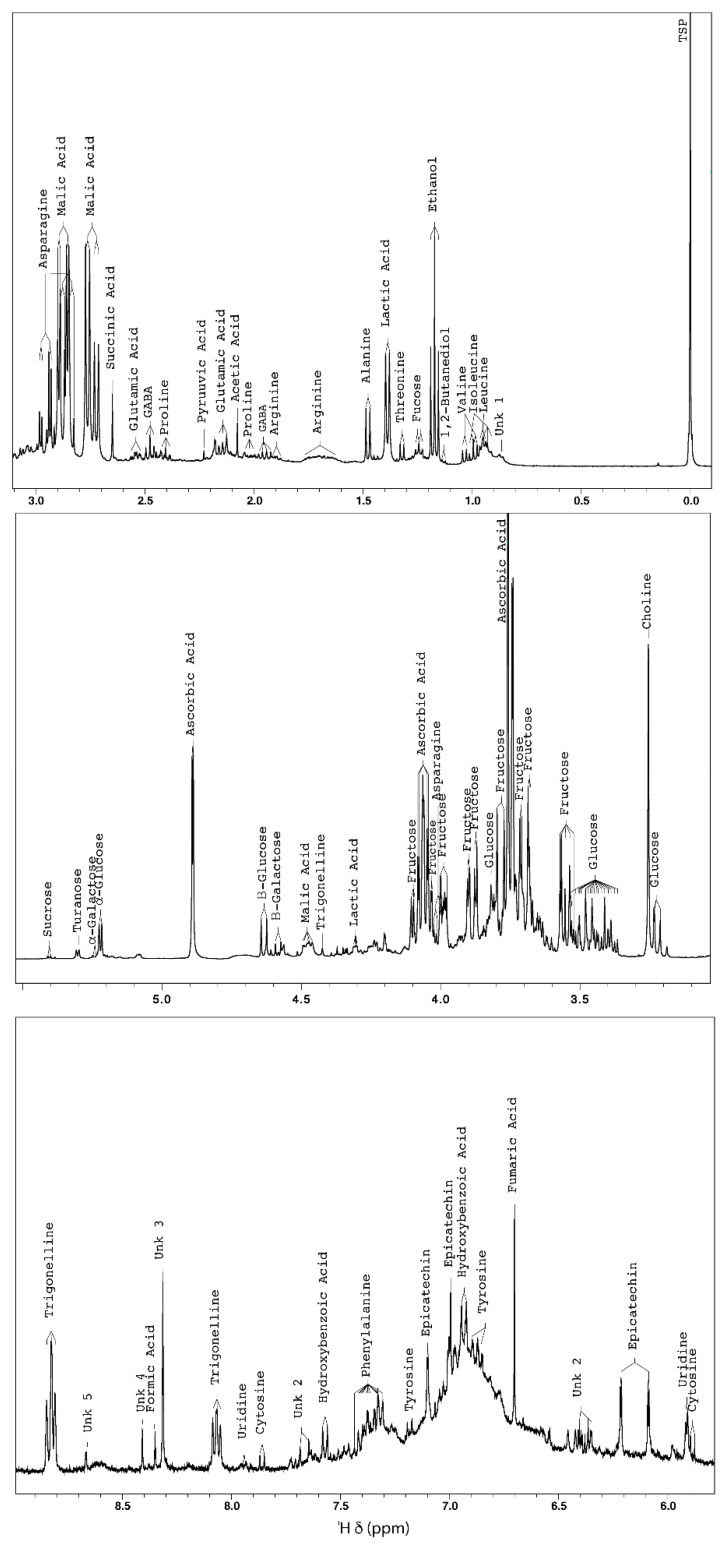
Representative ^1^H-NMR spectrum of acerola juice concentrate at δ 0.0–9.0 ppm showing characteristic signals for primary and specialized metabolites or compounds (400 MHz, 300 K, D_2_O, pH 3.35, 20 mM TSP).

The observation of the annotated 1D ^1^H-NMR spectrum of acerola concentrate (Figure 2) revealed that the most intense signals corresponded to the signals of ascorbic acid (d, 4.88 ppm- m, 4.06 ppm- m, 3.74 ppm).

Many signals of primary metabolites, i.e., amino acids, organic acids, and sugars, were also identified (Table 2, Figure 2). In the aliphatic region, the amino acid signals of leucine, isoleucine, valine, threonine, alanine, proline, arginine, glutamic acid, and asparagine were detected. In the same region, the organic acid signals of lactic, acetic, pyruvic, succinic, and malic acids were detected.

Nitrogenous compounds such as GABA and choline, an essential nutrient with structural, metabolic, and regulatory roles within the human body [31], were also detected and with notable resonance intensities.

The triplet at 1.17 ppm, corresponding to the CH_3_ group of ethanol, was also detected. The presence of ethanol in acerola concentrate is related to undesirable fermentation, which can be observed during the extraction and concentration of fruit juices [32]. 

Sugars were mainly represented by glucose (α and β) and sucrose, characterized by their anomeric protons at 5.22 ppm (d), 4.63 ppm (d), and 5.40 ppm (d), respectively. Signals from fructose and galactose (α and β), were also detected with low intensity. Turanose, a disaccharide, was annotated based on literature data [33].

However, the aromatic zone showed very weak signals characteristic of different molecules of different classes. Signals from nucleosides and nucleic bases, such as uridine and cytosine; phenolic compounds, such as hydroxybenzoic acid and epicatechin; the amino acids phenylalanine and tyrosine; and the alkaloid trigonelline [34] were detected. Phenolic acids and flavonoids are specialized metabolites that contribute mainly to the antioxidant properties of acerola [35]. Their antioxidant effect results from their ability to bind free radicals, donate hydrogen atoms or electrons, and catch metal cations [6]. Acerola has been shown to be richer in polyphenols than other fruits [36]. Trigonelline is a bioactive alkaloid, present in many fruits, with antioxidant properties and medicinal applications [37,38].

The present study identified a total of 36 metabolites in acerola juice concentrate, which is in global agreement with the literature for most compounds, but also complements previously published annotations. The contents of these metabolites may vary according to the origin of the concentrates. Indeed, Da Franca et al. [6], in their study on acerola fruits of different clones, showed that some metabolite contents are biomarkers of maturity stages. Indeed, ascorbic acid and choline seem to have higher contents in immature or partially mature fruit and are major signals in 1D ^1^H-NMR spectra of acerola juices [6]. The singlet peak of choline and doublet of ascorbic acid were well resolved and highly detectable on NMR spectra of acerola concentrate in the present experiment. Therefore, we went further and evaluated the resonance intensity of each of these metabolites of interest and the correlation between the intensity of these two metabolites. For this purpose, 117 acerola juice concentrates received between 2020 and 2022 were analyzed by 1D ^1^H-NMR, and the ascorbic acid and choline areas and their ratios (AA peak area/choline peak area) were calculated to evaluate the variations in their contents and relative contents. The mean of the 117 area ratios obtained was equal to 2.0, with a relative standard deviation of 13.8% (Table S1, see details in the Supplementary Materials). This meant that the ratio between the contents of the two metabolites was stable, which is in agreement with the results of Da Franca et al. in fruit [6]. 

We propose that the presence of one metabolite and the absence of the other, especially the presence of ascorbic acid and the absence of choline, may indicate that acerola concentrate has been supplemented with exogenous ascorbic acid. Thus, the simultaneous presence of both metabolites measured using 1D ^1^H-NMR can be used to evaluate the origin of ascorbic acid in acerola extracts, i.e., the presence of ascorbic acid alone or in much higher quantities than choline, or the opposite, in an acerola extract can be suspicious and linked to the addition of exogenous or synthetic ascorbic acid or choline in this extract. In conclusion, the ratio of these two metabolites can be used as a marker of the authenticity of acerola extracts.

#### 2.1.2. NMR Fingerprinting of Acerola-Based Food Supplements

By industrial processes, the concentrated juices of unripe acerola fruit are transformed into powder to be incorporated into food supplements. This transformation is carried out at a high temperature, which can cause a loss or reduction in the content of specific heat-sensitive metabolites, such as the key metabolite ascorbic acid [39]. Consequently, controlling these dried extracts to assess their quality is of the utmost significance.

In this work, we aimed to evaluate the application of ^1^H-NMR-based metabolomics in controlling the quality of acerola-based food supplements. For this purpose, targeted metabolomic profiling was performed to compare commercial samples to an in-house acerola reference powder to assess authenticity and differences in composition. 

All acerola-based food supplements used in this study were ordered online. Table S2 provides the contents listed on the supplement labels by the manufacturers and the galenic form of each product. First, the profile of the acerola reference powder and that of the concentrated juice were compared visually (Figure S1). Apart from the sugar region, the aliphatic and aromatic spectral regions exhibited fewer signals than those of concentrated juice. This was explained by the dilution of the acerola juice concentrate during industrial processing with drying support. Our reference powder was titrated with at least 17% (*w*/*w*) ascorbic acid. 

**Figure 3:**
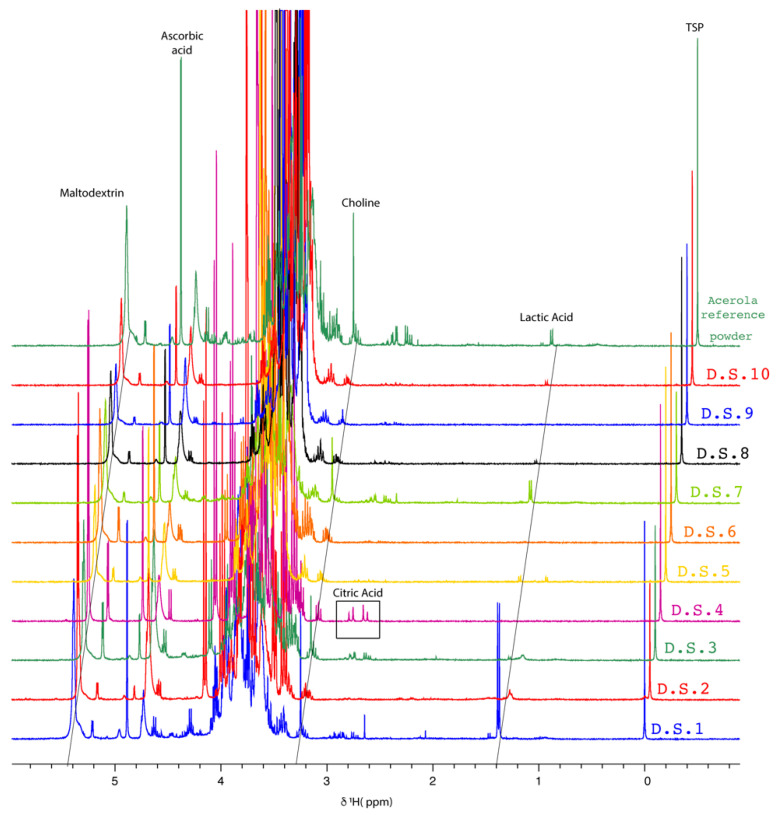
Stacked plot of 1D ^1^H-NMR spectra of each food supplement sample (400 MHz, 300 K, D_2_O, pH 3.35, 20 mM TSP).

To enable visual comparisons based on spectral profiles with respect to the acerola reference powder, Figure 3 includes stacked spectra of the food supplements tested. The ^1^H-NMR profiles of 4 out of 10 food supplements (D.S.1, 3, 5, and 7) were close to the acerola reference powder profile. In contrast, the remaining profiles showed different compositions. Indeed, food supplements 1, 3, 5, and 7 were the only samples to contain ascorbic acid and choline signals, as in the reference acerola powder. In addition, the aliphatic region of these samples included some signals of amino acids and organic acids. The aromatic region exhibited very-low-intensity signals of trigonelline (Figure S2). 

For all other spectra, except for sample D.S.4, no amino acid signals in the aliphatic region or characteristic signals in the aromatic region (Figure S2) could be detected. Only organic acid signals (lactic, acetic, and malic acids) were detected. In contrast to the other samples, D.S.4 was distinguished by the presence of three compounds, namely citric acid, sucrose (intense doublet at 5.40 ppm), and a putative flavonoid (5.85 ppm, 5.89 ppm, 7.03 ppm, data not shown). The three compounds were in accordance with the manufacturer’s specifications, with sugar, citric acid, and extract of *Citrus paradisi* seed. The *C. paradisi* seed contains flavonoids [40].

Furthermore, a broad and intense signal at 5.40 ppm was detected in the acerola reference powder and in all food supplement spectra except that of D.S.4. This signal corresponded to one signal of maltodextrin, the support used during the industrial process of drying the acerola concentrate to obtain the powder.

To verify the ratio of the ascorbic acid over choline in the processed powders, 50 samples of acerola powder, produced in-house, were analyzed, and the average value of the obtained ratios was equal to 2.15 ± 0.25 (Table S2). This value was in the defined range for juice concentrate. Then, the AA/choline ratios of each ^1^H-NMR spectrum obtained for the food supplements were measured (Table 2). According to the visual inspection of the spectra and Table 2, only three products (D.S.1, 5, and 7) had the expected ratio defined above for concentrated acerola juice. D.S.3 had a low ratio, which meant an unexplained low amount of ascorbic acid. In contrast, the other products had ratios more than ten times greater than the predefined ratio (2.00 ± 0.26), suggesting that the ascorbic acid in these products did not originate 100% from the concentrated acerola juice used.

In conclusion, the comparison of the NMR spectra showed significant qualitative differences. Only the D.S.1, 5, and 7 spectra seemed to contain signals from acerola powder. The absence of expected metabolites in the other samples suggested a low amount or absence of acerola powder in these products, and their ascorbic acid may have been of synthetic origin. Therefore, it was necessary to quantify the ascorbic acid in the different products and compare the contents obtained with the contents claimed by the manufacturers.

### 2.2. Quantitative Analysis of Ascorbic Acid in Acerola Juice by ^1^H-NMR 

#### 2.2.1. pH Optimization of the Acerola Sample

Ascorbic acid is a water-soluble molecule with two ionizable hydroxyl groups, and its gamma-lactone ring makes it highly reactive. Its chemical shifts (Figure 2) change with a varying pH, so we studied this dependence to prevent potential overlaps of the signal of interest with the signal of other metabolites in the acerola solution.

**Figure 4:**
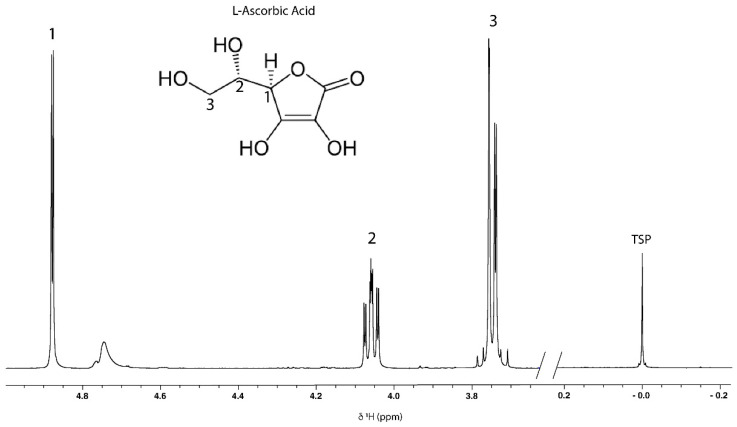
1D ^1^H-NMR spectra of L-ascorbic acid in D_2_O (400 MHz, 300K, pH 3.35, 20 mM TSP).

First, a 1D ^1^H-NMR spectrum of ascorbic acid (Tracert^®^, Sigma-Aldrich Chimie Merck—Saint-Quentin Fallavier, France) in D_2_O (Eurisotop, Gif sur Yvette, France) was recorded in order to assign the signals of its different protons (Figure 4). Peaks at around 4.88 ppm were assigned to H1 protons; those at 4.06 ppm to H2 protons; and those at around 3.74 to H3 protons of ascorbic acid. Because (OH) hydroxy protons interchange rapidly with deuterated water, they are not characterized by distinct resonances [41].

The comparison of ascorbic acid and acerola spectra (Figure S3) showed that the H1 proton of ascorbic acid was the only proton whose chemical shift was isolated and could be used for quantification. Thus, the pH optimization was carried out using a sample of the acerola reference powder to check the position of the H1 signal of interest in relation to the other signals that could be found in the spectra of the acerola reference powder.

**Figure 5:**
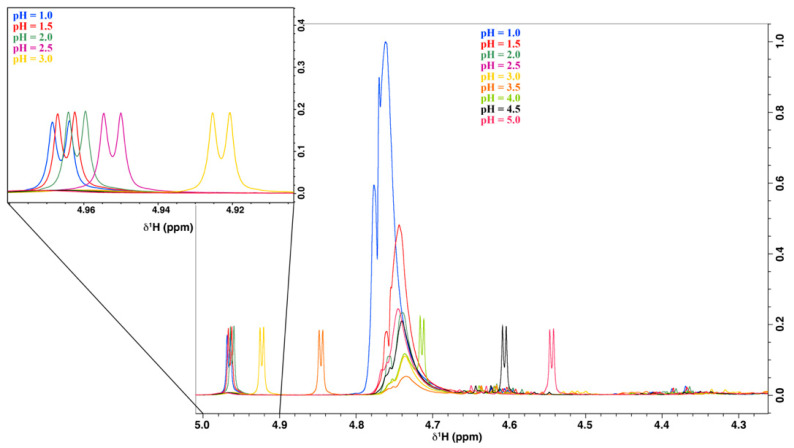
Variations in the ascorbic acid H1 chemical shift in acerola powder solution as a function of pH (400 MHz, 300 K, pH 1.0 to 5.0, D_2_O).

The pH optimization was performed with respect to the chemical shift of the H1 signal of ascorbic acid. Nine solutions of acerola reference powder with the pH adjusted to 1.0, 1.5, 2.0, 2.5, 3.0, 3.5, 4.0, 4.5, and 5.0 were prepared and used to record 1D ^1^H-NMR spectra. The chemical shift of the H1 proton decreased with an increasing pH (Figure 5), in agreement with published results about AA protonation equilibrium [42]. At a pH of 1 to 3, the H1 resonance signals overlapped with the maltodextrin signal at 4.94–4.98 ppm. At pH 4, the H1 resonance signals were at about 4.71 ppm, but the presaturation pulse sequence used to suppress the water signal caused distortion in this region, which may have impacted quantification. At a pH above 4, the H1 signals overlapped with the sugar signals. Therefore, at the higher pH values, the H1 signal could be used to quantify ascorbic acid. The best position of the peak, allowing it to be isolated and quantifiable, was at 4.92 ppm, which was obtained at pH 3.5. After a fine adjustment, the pH value retained was 3.35.

#### 2.2.2. NMR Acquisition Conditions for Quantitative Analysis of Ascorbic Acid

Several water suppression pulse sequences are available in NMR, mainly for biomolecular research [36], and their selection should be carried out with care since it could impact the spectrum quality and quantification result. In NMR-based metabolomics, two sequences are commonly used: the simplest presaturation pulse sequence (zgpr in Bruker library, PRESAT in Varian library, Proton with presaturation in JEOL library) and 1D-NOESY pulse sequence. The 1D-NOESY sequence was chosen for its robustness [43] and because it seems to be the sequence most well-adapted for NMR-based metabolomics investigations [44].

The parameters of the pulse sequence were carefully considered for the accuracy of the quantification: pulse length and mixing time, relaxation delay, number of scans, and acquisition time. The 90° pulse length was optimized on the H1 AA signals of ten ascorbic acid solutions, and the resulting 90° pulse length variation was 0.11 µs; therefore, the 90° pulse length was fixed equal to 14 µs for all 1D ^1^H acquisitions. 

In order to achieve high precision in the quantitative analysis, the delay between pulses, or the repetition rate, must be five times the longest T1 for a maximum error of 1%. T1 values were measured from the inversion recovery experiments (Table 3). Under the conditions of our study, the H1, H2, and H3 signals had a T1 lower than 4 s; therefore, the relaxation delay or repetition rate (d1) was set at 20 s to ensure that all signals used for the quantitative measurements relaxed completely between pulses and to achieve a maximum error related to T1 below 1%.

To select the best values for scan number and acquisition time, twelve 1D ^1^H-NMR spectra of a 9 mg/mL solution of ascorbic acid were acquired with variations in the values of these two parameters (Table 4).

A scan number of 16 and an acquisition time of 4 s were selected as a compromise between the acquisition of spectra with adequate sensitivity and resolution (SNR) and a short experiment time (8 min per spectrum). 

A calibration reference had also to be chosen for quantitative NMR analysis. In NMR, the peak area of fully relaxed nuclear spins is proportional to the number of nuclei generating that peak [45]. The number of nuclei is directly linked to the molar concentration of the molecule in the NMR tube, from which the concentration and the amount of the analyte can be derived. Various referencing methodologies have been developed for qNMR, including internal and external references. Bharti and Roy provided a detailed review of different methods, including their advantages and disadvantages [46].

For this study, because AA is an unstable molecule, we chose the relative quantification by external calibration curve method using a stock solution freshly prepared from standard ascorbic acid (Tracert^®^ with high purity). The reference solution and the samples were analyzed under exactly the same NMR conditions.

#### 2.2.3. Method Validation

The method was validated according to the Eurolab technical report [47].

- Precision

The precision (reproducibility) of the qNMR method was evaluated by an intra-day repeatability test. Ten replicate measurements of the same AA solution were recorded. The relative standard deviation (RSD) value of the ascorbic acid H1 area was 0.50% (Table S3) compared to the RSD obtained for TSP (0.66%) in the same solutions, which indicated that the method had very good precision.

- Specificity

The specificity of the developed method was verified using the ascorbic acid H1 area (4.88 ppm) in ^1^H-NMR spectra of acerola powder representative samples. We demonstrated that this area did not contain any other peaks based on the sensitivity of this peak to pH variations, i.e., by observing the variations in the chemical shifts as a function of the pH, no other peak was detected. It was also verified on 2D spectra. Thus, this peak was ideal for quantification, and its shape was symmetrical without any signal interference observed in the evaluated region (Figure S2).

- Linearity

**Figure 6:**
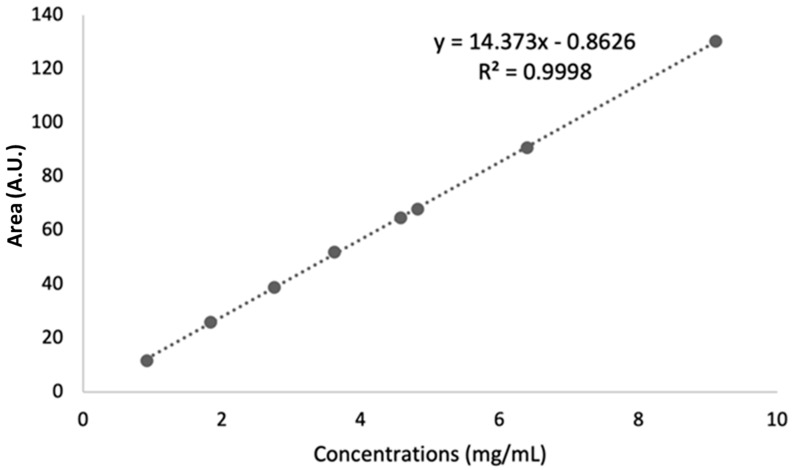
AA calibration curve. Concentration range 0.91–9.11 mg/mL. A.U.: arbitrary unit.

To check the linearity of the developed method, a series of eight synthetic samples containing AA over the range of 0.91–9.11 mg/mL was used to plot the calibration curve (Figure 6). The concentration ranges were chosen based on the standard ascorbic acid content of commercialized acerola-based food supplements. The calibration data are summarized in Table 5. The coefficient of determination (R^2^) was greater than 0.999, showing a high linear response in the concentration range studied.

- Analytical Limits of Detection and Quantification

There are many definitions of LOD; we chose to calculate it from a calibration curve using Equation (1) [48]. The obtained value of the LOD was 0.05 mg/mL. The limit of quantification (LOQ) was considered equal to three times the LOD (LOQ = 0.15 mg/mL).

LOD (mg/mL) = (3S_y/x_ + intercept)/slope (1)

- Ascorbic acid stability

The stability of AA was determined using ten NMR tubes with different AA concentrations. All tubes were kept in the sample changer at 4 °C and were analyzed randomly once a day for seven days. For each spectrum, the area under the curve of the H1 peak was integrated automatically using NMRProcFlow [49]. The relative standard deviation (RSD) for each tube is reported in Table 6. A maximum RSD of 6.01% was observed, and this deviation was more critical for the tubes with lower amounts of AA. 

**Figure 7:**
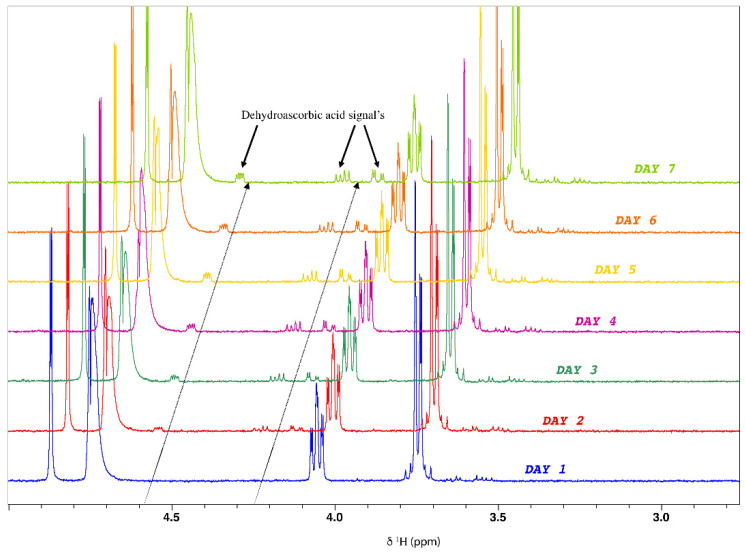
Evolution of the ascorbic acid spectrum as a function of time with the appearance of dehydroascorbic acid signals (400 MHz, 300 K, D_2_O, pH 3.35, 20 mM TSP).

Figure 7 shows the superposition of the spectra obtained for tube 1, which had the lowest AA concentration. From day 2, the signals of dehydroascorbic acid (DHA) were detected and their intensity increased as a function of time. DHA is an oxidized form of ascorbic acid [50]. Its quantity increased as a function of time. Thus, the variations observed in the AA concentration (Table 6) were explained by its oxidation and transformation into DHA. 

Regarding the results of the method validation, parameters such as precision, specificity, linearity, LOD, LOQ, and stability were in acceptable ranges. This method is appropriate for the quantification of AA in acerola-based preparations for routine analysis. 

#### 2.2.4. Application: Ascorbic Acid Quantification in Commercial Food Supplements

To validate the NMR protocol, samples from 10 commercial food supplements were subjected to the quantitative analysis of AA using the developed 1D-^1^H qNMR method. The pH was adjusted to 3.35 for all samples. A spectrum of each sample is represented in Figure 3. On all spectra of pH-adjusted samples, the AA H1 peak of interest was well resolved, and no overlapping occurred. The results of the qNMR analyses were plotted against those declared by the manufacturers and are reported in Table 7.

The results obtained by ^1^H qNMR were close to the values declared on the labels, except for D.S.2 and D.S.3. The difference could be explained by the fact that the AA contents declared on the label were the contents of the acerola powder used to manufacture the product and not the content of the final product. Hence, the values indicated in Table 7 (AA% in label) are the theoretical values expected in the final products.

For D.S.2, the quantifiable peak was poorly resolved with a low SNR ratio, which excluded the NMR quantification of AA in this product. For D.S.3, the quantity obtained corresponded to half the expected value, in agreement with the low value of 0.96 for the ratio of areas (AA/choline) (Table 2), which may be explained by the exogenous origin of the choline.

In conclusion, this ^1^H qNMR method can be used by manufacturers of acerola-based food supplements to quantify ascorbic acid. The NMR experimental time of 8 min is short compared to chromatography-based methods (e.g., 14 min [51]). Despite the high LOD of 0.05 mg/mL compared to chromatography-based methods (e.g., 0.53 µg/mL [51]), this method is of great interest for acerola-based food supplements because the optimized working range is between 0.91 mg/mL and 9.11 mg/mL. Moreover, the developed method allows the simultaneous verification of acerola metabolomic profiles and the quantification of ascorbic acid with a light sample preparation, dilution and pH adjustment, and in a short time. Such an approach could also be pertinent for AA-containing food supplements other than those based on acerola, such as those based on camu-camu (*Myrciaria dubia*), another tropical fruit with a high AA content [5].

## 3. Materials and Methods

### 3.1. Sample Details

The concentrated acerola juices were purchased from EVEAR Extraction company suppliers in Brazil. They were transformed into acerola powder as part of the company activity.

All acerola-based food supplements were purchased online (SantéDiscount.com, accessed on 15 September 2021). Table S2 lists the components disclosed by supplement manufacturers on their packages.

### 3.2. Reagents and Materials

The chemical reagents used were of analytical grade. Deuterium oxide (D_2_O) was purchased from Eurisotop (Gif sur Yvette, France). Hydrochlorid acid 37% (HCl), sodium hydroxide (NaOH), 3-(trimethylsilyl)-2,2,3,3-tetradeuteropropionic acid sodium salt (TSP), and ascorbic acid Tracert ^®^ were ordered from Sigma-Aldrich Chimie Merck (Saint-Quentin Fallavier, France).

### 3.3. Instruments and Software

The ^1^H-NMR experiments were performed on a Bruker Avance III 400 spectrometer equipped with a 5 mm z-gradient ATMA-BBO probe operating at 300 K and a sample changer (SampleCase cooled) that was used at 4 °C. TopSpin V3.5 pl 6 software (Bruker Biospin, Wissembourg, France) was used for NMR data acquisition, processing, and analysis, and its IconNMR module controlled the automation of the acquisition, i.e., locking, tuning, matching, and shimming.

Sample temperature was regulated at 300 K (27 °C) with an interval of 2 min for setting the temperature before data acquisition. Automatic tuning and matching of the probe was carried out for each sample. Samples were not spun.

The Bruker Titration pH unit (BTpH, Bruker, Karlsruhe, Germany) was used to quickly and precisely adjust the pH of samples with an HCl or NaOH 1 M solution. 

NMRProcFlow [49], an open-source software for NMR spectra processing, was used for the automatic alignment of the peaks in the areas of interest using the cluster-based peak alignment (CluPA) algorithm [52] and for the peak integration of the spectra for the calculation of AA area/choline area ratios and the quantification of ascorbic acid using two variable-size buckets, one centered at the doublet of the AA H1 peak and the second at the singlet of choline.

### 3.4. General Procedure of ^1^H-NMR Acquisition and Processing

^1^H-NMR spectra were recorded using a 1D NOESY (noesygppr1d) pulse sequence. Acquisition parameters were: 64K data points, 8223 Hz (20 ppm) spectral width, 4 dummy and 16 scans, a recycle delay of 20 s, 14 µs for the hard pulse, 10 ms mixing time, and a fixed value for receiver gain for all samples. The water signal was suppressed by presaturation using continuous irradiation during relaxation delay. Spectra were locked and shimmed automatically using IconNMR. A line-broadening function of 0.3 Hz was applied automatically to all spectra for Fourier transformation with zero filling (X2). Finally, spectra were automatically phased, baseline corrected, and referenced to TSP at 0.0 ppm with TopSpin.

The quantification was performed on the ^1^H-NMR signal of ascorbic acid H1 (doublet: d). Using NMRProcFlow, all spectra were aligned to the peak of interest using the CLuPa algorithm. The integration was carried out automatically in the same way for all spectra. The data matrix was exported, and the areas obtained were used to quantify ascorbic acid.

### 3.5. Metabolomic Profiling of Acerola Juice Concentrate

An NMR sample was prepared for metabolomic profiling by dissolving 59.20 mg of acerola juice concentrate in 800 µL of D_2_O (with 20 mM TSP). Its pH was adjusted to 3.35 by adding 1.80 µL of hydrochloric acid 1M. An amount of 600 µL was transferred to an NMR tube.

The 1D ^1^H NOESY NMR spectrum was recorded as described above (4.4) but with 512 scans.

The spiking of seven-teen acerola juice concentrates with one commercial compound at once followed by pH adjustment was carried out to confirm most annotations. To remove ambiguity about the attribution of specific signals, 2D NMR experiments were recorded. ^1^H -^1^H TOCSY spectra were acquired with 256 indirect points, 2048 direct points (spectral width of 12 ppm in both dimensions), 32 scans, and a recycle time of 2 s, using the dipsi2esgpph pulse program. The ^1^H -^13^C HSQC spectrum was also recorded with 512 indirect points, 2048 direct points (spectral width of 16 ppm for direct dimension (^1^H) and 165 ppm for indirect dimension (^13^C)), 32 scans, and a recycle time of 2 s, using hsqcetgpsisp2.4 pulse program. Finally, the ^1^H -^13^C HMBC spectrum was recorded with 512 indirect points, 2048 direct points (spectral width of 20 ppm for direct dimension (^1^H) and 220 ppm for indirect dimension (^13^C)), 32 scans, and a recycle time of 2 s, using hmbcgplpndqf pulse program. Long-range J(CH) was fixed at 10 Hz and low-range J(CH) at 145 Hz. Spectra were locked and shimmed automatically using IconNMR software. Finally, spectra were manually phased, baseline corrected, and referenced to TSP at 0.0 ppm. 

### 3.6. pH Optimization 

A stock solution of acerola reference powder was prepared by dissolving 134.99 mg in 8 mL of D_2_O (with 20 mM TSP). Then, the stock solution was divided into 9 samples. The pH of each sample was adjusted using the Bruker titration unit (BTpH, Bruker) to a single value to cover a range between 1 and 5 ± 0.02. An amount of 600 µL of each pH-adjusted solution was transferred to an NMR tube. All NMR spectra were recorded using the general procedure detailed above (see Section 3.4).

### 3.7. T1 Measurement

To measure the T1 of ascorbic acid signals, an NMR sample was prepared from a 6.17 mg/mL ascorbic acid stock solution. The inversion recovery pulse (T1ir) was used to measure the longitudinal relaxation delay τ. Data were fitted to the exponential Equation (2), where *A* is the area of each proton resonance at inversion delay *T* and *A*_0_ at the equilibrium state. *P* is a constant. The list delay (*T*) was: 0.1, 0.5, 1.0, 1.4, 1.8, 2.2, 2.5, 3.0, 3.5, 4.0, 4.5, 5.0, 6.0, 8.0, 10.0, and 12.0 s.

(2) A=A0+P e−τT

### 3.8. Calibration Curve

Eight standards were obtained by dilution in the range of 0.91–9.11 mg/mL from two freshly prepared stock solutions of Tracert ^®^ ascorbic acid (Sigma-Aldrich Chimie Merck, Saint-Quentin Fallavier, France) at 11.08 mg/mL and 14.72 mg/mL, respectively. After pH adjustment to 3.35, their NMR spectra were recorded according to the general procedure detailed above (see Section 3.4). Peak integration was carried out automatically in the same way for all spectra using NMRProcFlow. The data matrix was exported, and the areas obtained were used to quantify ascorbic acid. Then, the areas were plotted against the concentrations for calibration.

### 3.9. NMR Fingerprinting of Acerola-Based Food Supplements and Quantification of Ascorbic Acid

For NMR sample preparation, tablets were ground and homogenized with a mortar before analysis, and capsules were emptied. About 15 mg of powder for each sample was dissolved in 800 µL D_2_O supplemented with 20 mM TSP. The pH was adjusted by automatically adding the necessary volumes of acid (HCL-1 M) or base (NaOH-1 M) with BTpH. An amount of 600 µL of each pH-adjusted solution was transferred to an NMR tube.

The 1D ^1^H NOESY NMR spectrum was recorded as described above (see Section 3.4). 

The quantification was performed on the ^1^H-NMR signal of AA H1 (doublet). Using NMRProcFlow [49], all spectra were aligned to the peak of interest. The integration was carried out automatically with a variable-size bucket centered at the doublet of the AA H1 peak in the same way for all spectra. The data matrix was exported, and the areas obtained were used to quantify AA. The spectra of ascorbic acid solutions used to draw the calibration curve were treated in the same series of food supplement powders.

## 4. Conclusions

In this work, we aimed to develop and implement a rigorous method for the quality control of food supplements containing acerola. Analyzing the metabolomic profile of concentrated acerola juice enabled us to identify the AA/choline ratio as an authenticity marker of acerola extracts. Given the low prices of synthetic ascorbic acid (EUR 5 to 7 /kg) and choline (EUR 5 to 10 /kg), the production of a false product with good ascorbic content at a low cost compared to the price of acerola concentrate juice (EUR 20 to 30 /kg) is possible. In fact, the observation of the ^1^H-NMR-based metabolomic profile (detection of some signals of amino acids, organic acids, and trigonelline) and of the ratio between the amounts of AA and choline enabled us to identify the presence of exogenous ascorbic acid and detect such fraudulent manipulations. 

Furthermore, the qNMR method developed in this study allows the quantification of ascorbic acid in concentrated acerola juice, spray-dried acerola powder, and acerola-based formulations using a simple NMR experiment. The results for the validation parameters such as linearity, limit of detection (0.05 mg/mL), and limit of quantification (0.15 mg/mL) were in an acceptable range. Due to the rapid sample analysis time (8 min), this method can be applied for the routine analysis of numerous samples of acerola-based food supplements.

## Figures and Tables

**Table 1:** Compounds and ^1^H chemical shifts identified by 400 MHz 1D ^1^H-NMR for acerola juice concentrate (300 K, D_2_O, pH 3.35, 20 mM TSP).

Compounds	Chemical Shift (ppm) Multiplicity (J in Hz)	MSI Status *	Identification Confirmation
Isoleucine	0.93 (t, J = 7.56); 1.00 (d, J = 6.64)	1	Spike, TOCSY correlation
Leucine	0.95 (t, J = 4.94)	1	TOCSY correlation
Valine	0.98 (d, J = 7.02); 1.03 (d, J = 7.02)	1	TOCSY correlation
1, 2-Butanediol	1.13 (d, J = 6.08)	1	Spike, TOCSY correlation
Ethanol	1.17 (t, J = 7.08)	1	TOCSY correlation, ^1^H-^13^C HSQC
Fucose	1.24 (d, J = 6.48); 1.25 (d, J = 5.80)	2	TOCSY correlation
Threonine	1.32 (d, J = 6.60)	1	TOCSY correlation, ^1^H-^13^C HSQC
Lactic acid	1.38 (d, J = 6.96); 4.31 (q, J = 1.56)	1	TOCSY correlation, ^1^H-^13^C HSQC
Alanine	1.47 (d, J = 7.28)	1	Spike, TOCSY correlation
Arginine	1.69 (m); 1.89 (m)	1	Spike, TOCSY correlation
GABA	1.94 (q, J = 7.48); 2.47 (t, J = 7.32)	1	TOCSY correlation
Proline	2.01 (m); 3.55 (m)	1	TOCSY correlation
Acetic acid	2.07 (s)	2	-
Glutamic acid	2.14 (m); 2.54 (m)	1	Spike, TOCSY correlation
Pyruvic acid	2.23 (s)	2	-
Succinic acid	2.64 (s)	1	Spike
Malic acid	2.74 (dd, J = 7.46, J = 16.27); 2.87 (dd, J = 4.50, J = 16.15)	1	TOCSY correlation, ^1^H-^13^C HSQC
Asparagine	2.86 (dd, J = 7.76, J = 16.89); 2.96 (dd, J = 4.30, J = 16.99); 4.01 (dd, J = 4.46, J = 7.38)	1	Spike, TOCSY correlation, ^1^H-^13^C HSQC
β-glucose	3.23 (dd, J = 8.00, J = 7.92); 4.63 (d, J = 7.96)	1	Spike, TOCSY correlation, ^1^H-^13^C HSQC
Choline	3.25 (s)	1	TOCSY correlation, ^1^H-^13^C HSQC and ^1^H-^13^C HMBC correlation
Ascorbic acid	3.74 (m); 4.06 (m); 4.89 (d, J = 1.88)	1	Spike, TOCSY correlation, ^1^H-^13^C HSQC
Fructose	4.03 (m)	1	Spike, TOCSY correlation, ^1^H-^13^C HSQC
Trigonelline	4.42 (s); 8.07 (m); 8.83 (m); 9.12 (s)	1	TOCSY correlation
β-galactose	4.57 (d, J = 7.84)	1	Spike
α-glucose	5.22 (d, J = 3.76)	1	Spike, TOCSY correlation, ^1^H-^13^C HSQC
α-galactose	5.25 (d, J = 3.96)	1	Spike
Turanose	5.30 (d, J = 3.84)	2	-
Sucrose	5.40 (d, J = 3.80)	1	Spike, TOCSY correlation, ^1^H-^13^C HSQC
Cytosine	5.89 (d, J = 7.56); 7.86 (d, J = 8.64)	2	TOCSY correlation
Uridine	5.91 (d, J = 3.60); 7.94 (d, J = 4.44)	2	TOCSY correlation
Epicatechin	6.09 (d, J = 2.22); 6.21 (d, J = 2.22); 6.99 (Br.s); 7.10 (d, J = 1.76)	2	TOCSY correlation
Fumaric acid	6.70 (s)	2	-
Tyrosine	6.88 (m); 7.18 (m)	1	Spike, TOCSY correlation
Hydroxybenzoic acid	6.93 (d, J = 8.80); 7.57 (d, J = 8.64)	2	TOCSY correlation
Phenylalanine	7.37 (m)	1	Spike, TOCSY correlation, ^1^H-^13^C HSQC
Formic acid	8.34 (s)	1	Spike
Unk 1	0.86 (d, J = 6.11); 3.11 (N.D.); 3.76 (N.D.)	4	TOCSY correlation
Unk 2	6.37 (m); 7.66 (d, J = 6.36)	4	TOCSY correlation
Unk 3	8.31 (s)	4	-
Unk 4	8.40 (s)	4	-
Unk 5	8.66 (d, J = 1.36)	4	-

GABA: gamma-amino butyric acid; Unk: unknown compound. Multiplicity: s = singlet, d = doublet, dd = doublet of doublets, t = triplet, q = quartet, m = multiplet. Br.s. = broad singlet; N.D.: not defined. * MSI level of identification according to Sumner et al. [30]. Spike: addition of commercial compounds in acerola concentrate juice with pH adjustment at 3.35.

**Table 2:** Calculated ratio (AA area/choline area) for ^1^H-NMR spectra from food supplements.

Food Supplements	Ratio (AA Area/Choline Area)	Remarks
D.S.1	2.23	-
D.S.2	N.C.	AA detected but not quantifiable
D.S.3	0.96	-
D.S.4	N.C.	Choline not detected
D.S.5	2.21	-
D.S.6	23.13	-
D.S.7	1.83	-
D.S.8	26.79	-
D.S.9	10.39	-
D.S.10	29.69	-

N.C. = not calculated.

**Table 3:** T1 measurement of AA in D_2_O (400 MHz, 6.17 mg/mL, D_2_O, pH 3.35, 300 K).

Protons	Chemical Shift (ppm)	T1 Values (s)
H1	4.850	3.417
H2	4.016	3.315
H3	3.705	1.047

**Table 4:** Impact of scan number (NS) and acquisition time (Aq time) on AA peak area and signal/noise ratio (SNR) in ^1^H-NMR spectra of an ascorbic acid solution.

Experiment n°	NS	D1 (s)	Aq Time (s)	Experiment Time (min)	H1 Peak Area (a.u.)	SNR
1	4	20	2	2.56	31.58	628.96
2	4	20	4	3.12	31.68	608.34
3	4	20	8	3.44	31.78	617.52
4	8	20	2	4.24	63.10	858.63
5	8	20	4	4.48	63.05	829.83
6	8	20	8	5.36	63.56	859.87
7	16	20	2	7.20	126.00	1104.08
8	16	20	4	8.00	126.29	1170.88
9	16	20	8	9.20	126.40	1192.31
10	32	20	2	13.13	252.81	1716.74
11	32	20	4	14.25	253.20	1617.95
12	32	20	8	16.49	254.53	1665.14

a.u.: arbitrary unit.

**Table 5:** Regression parameters of the method.

Slope	Intercept	R^2^	S_y/x_ *
14.373	−0.8626	0.9998	0.5387

* S_y/x_, standard error for regression.

**Table 6:** Evaluation of AA stability over time.

NMR Tube	AA Concentration (mg/mL)	RSD (%)
1	0.742	6.01%
2	1.485	4.17%
3	2.228	2.98%
4	2.238	3.48%
5	2.971	2.32%
6	3.357	2.12%
7	3.713	2.06%
8	4.456	1.62%
9	4.476	1.50%
10	5.199	1.59%

**Table 7:** Determination of AA in 10 food supplements measured in duplicate by the ^1^H qNMR method and comparison with the values declared by manufacturers (label).

Food Supplement	AA% in Label (*w*/*w*)	AA% Measured Using ^1^H qNMR (*w*/*w*) (mean ± SD, n = 2)
D.S.1	17.50%	15.28 ± 0.12%
D.S.2	1.60%	N.Q.
D.S.3	8.25%	4.10 ± 0.0%
D.S.4	15.87%	14.09 ± 0.13%
D.S.5	10%	9.77% ± 0.33%
D.S.6	12.50%	11.90 ± 1.18%
D.S.7	11.33%	9.35 ± 0.09%
D.S.8	7.89%	8.22 ± 0.06%
D.S.9	6.66%	6.65 ± 0.62%
D.S.10	7.50%	7.04 ± 0.45%

N.Q. = detected but not quantifiable.

## Data Availability

Representative metabolomic spectra, data, and metadata of this article are available at recherche.data.gouv.fr open repository (https://entrepot.recherche.data.gouv.fr/dataverse/inrae) under study identifier 10.57745/CPZTEW (https://doi.org/10.57745/CPZTEW) (all accessed on 6 August 2022).

## Supplementary Materials

The following are available online at https://www.mdpi.com/article/10.3390/molecules27175614/s1, Table S1: Calculated AA area/choline area ratio for ^1^H-NMR spectra of 117 samples of acerola concentrate juice, Table S2: Ingredients listed on the manufacturer labels, Table S3: Calculated AA area/choline area ratio for ^1^H-NMR spectra of 50 samples prepared from acerola powder, Table S4: Repeatability of qNMR method based on ^1^H-NMR, Figure S1: 1D ^1^H-NMR spectra of acerola concentrate juice (blue) and acerola reference powder (red), Figure S2: Zoom-in on signals observed in the aromatic region of food supplement ^1^H-NMR spectra, Figure S3: Comparison between spectra of ascorbic acid and the sample of solution prepared from acerola reference powder.

## Sample Availability

All the samples considered in this work are available from the authors upon request.
