# Supplementary material for: Development, Validation, and Use of 1H-NMR Spectroscopy for Evaluating the Quality of Acerola-Based Food Supplements and Quantifying Ascorbic Acid
Source: Molecules. 2022 Aug 31;27(17):5614. doi: 10.3390/molecules27175614 (PMC9458237; doi:10.3390/molecules27175614)
Supplement: Supplementary file 1 [file molecules-27-05614-s001.zip › molecules-1880637-supplementary.pdf]

Article title: Development, validation, and use of ^{1}H-NMR spectroscopy for evaluating the quality of acerola-based food supplements and quantifying ascorbic acid

Authors: Asma Bourafai-Aziez, Daniel Jacob, Gwladys Charpentier, Emmanuel Cassin, Guillaume Rousselot, Annick Moing and Catherine Deborde

Table S1. Calculated (AA area/Choline area) ratio for ^{1}H-NMR spectra of 117 samples of acerola concentrate juice

Sample code	AA area B4_8905	Choline area B3_2595	Ratio (AA area/ Choline area)
**Acerola EC 2021139LC01-E1_2**	117.96	52.96	2.228
**Acerola EC 2021139LC01-E2_2**	96.96	43.66	2.221
**Acerola EC 2021139LC02-E1_2**	111.55	51.70	2.158
**Acerola EC 2021139LC02-E2_2**	128.62	59.60	2.158
**Acerola EC 2021174LC01-E1_2**	114.65	56.95	2.013
**Acerola EC 2021174LC01-E2_2**	137.49	68.60	2.004
**Acerola EC 2021189LC01-E1_2**	126.72	64.59	1.962
**Acerola EC 2021189LC01-E2_2**	126.71	64.21	1.973
**Acerola EC 2021202LC01-E1_2**	136.79	66.63	2.053
**Acerola EC 2021202LC01-E2_2**	142.14	69.60	2.042
**Acerola EC 20190-1 E1_2**	119.69	60.52	1.978
**Acerola EC 20190-1 E2_2**	117.15	58.75	1.994
**Acerola EC 20197-1 E1_2**	138.29	72.33	1.912
**Acerola EC 20197-1 E2_2**	92.91	48.44	1.918
**Acerola EC 20197-2 E1_2**	96.37	46.60	2.068
**Acerola EC 20197-2 E2_2**	111.99	53.60	2.089
**Acerola EC 2021006LC01-E1_2**	94.14	48.41	1.945
**Acerola EC 2021006LC01-E2_2**	102.61	52.69	1.947
**Acerola EC 2021006LC02-E1_2**	87.86	44.60	1.970
**Acerola EC 2021006LC02-E2_2**	106.21	53.64	1.980
**Acerola EC 2021013LC01-E1_2**	96.86	54.86	1.765
**Acerola EC 2021013LC01-E2_2**	108.22	61.02	1.773
**Acerola EC 2021013LC02-E1_2**	103.23	57.76	1.787
**Acerola EC 2021013LC02-E2_2**	111.15	62.11	1.789
**Acerola EC 2021042LC03-E1_2**	86.61	55.47	1.561
**Acerola EC 2021042LC03-E2_2**	117.39	75.28	1.559
**Acerola EC 2021056LC01-E1_2**	107.97	44.93	2.403
**Acerola EC 2021056LC01-E2_2**	106.04	44.20	2.399

**Acerola EC 2021069LC01-E1_2**	92.50	42.37	2.183
**Acerola EC 2021069LC01-E2_2**	115.93	53.12	2.182
**Acerola EC 2021070LC01-E1_2**	102.51	56.02	1.830
**Acerola EC 2021070LC01-E2_2**	128.48	70.29	1.828
**Acerola EC 2021076LC02-E1_2**	95.93	43.46	2.207
**Acerola EC 2021076LC02-E2_2**	119.06	53.60	2.221
**Acerola EC 2021077LC02-E1_2**	86.81	39.62	2.191
**Acerola EC 2021077LC02-E2_2**	110.72	50.74	2.182
**Acerola EC 2021096LC03-E1_2**	91.26	46.59	1.959
**Acerola EC 2021096LC03-E2_2**	87.59	44.49	1.969
**Acerola EC 2021132LC01-E1_2**	159.56	81.76	1.952
**Acerola EC 2021132LC01-E2_2**	127.81	65.28	1.958
**Acerola EC 2021223LC01-E1_2**	145.11	86.41	1.679
**Acerola EC 2021223LC01-E2_2**	116.56	68.57	1.700
**Acerola EC 2021274LC02-E1_2**	117.31	69.10	1.698
**Acerola EC 2021274LC02-E2_2**	121.46	71.69	1.694
**Acerola EC 2021288LC01-E1_2**	106.02	62.38	1.700
**Acerola EC 2021288LC01-E2_2**	106.71	63.49	1.681
**Acerola EC 2021302LC01-E1_2**	128.33	71.24	1.801
**Acerola EC 2021302LC01-E2_2**	116.17	64.15	1.811
**Acerola EC 2021309LC01-E1_2**	125.04	65.70	1.903
**Acerola EC 2021309LC01-E2_2**	138.24	72.08	1.918
**Acerola EC 2021323LC01-E1_2**	146.81	80.24	1.830
**Acerola EC 2021323LC01-E2_2**	123.68	68.01	1.818
**Acerola EC 2021323LC02-bis-E1_2**	143.16	77.47	1.848
**Acerola EC 2021323LC02-bis-E2_2**	152.26	82.45	1.847
**Acerola EC 2021363LC01-E1_2**	106.71	47.51	2.246
**Acerola EC 2021363LC01-E2_2**	116.17	52.91	2.196
**Acerola EC 2021363LC02-E1_2**	97.76	44.17	2.213
**Acerola EC 2021363LC02-E2_2**	130.41	59.18	2.203
**Acerola EC 2022040LC03-E1_2**	110.94	47.67	2.327
**Acerola EC 2022040LC03-E2_2**	129.82	55.76	2.328
**Acerola EC 2022040LC04-E1_2**	102.21	43.75	2.336
**Acerola EC 2022040LC04-E2_2**	107.64	45.99	2.341
**Acerola EC 2022041LC01-E1_2**	112.39	43.83	2.564

**Acerola EC 2022041LC01-E2_2**	96.47	37.51	2.572
**Acerola EC 2022041LC02-E1_2**	101.10	37.24	2.715
**Acerola EC 2022041LC02-E2_2**	156.56	58.14	2.693
**Acerola EC 2022091LC02-E1_2**	125.32	55.45	2.260
**Acerola EC 2022091LC02-E2_2**	116.21	51.31	2.265
**Acerola EC 2022101LC01-E1_2**	112.76	53.09	2.124
**Acerola EC 2022101LC01-E2_2**	141.67	66.73	2.123
**Acerola EC 2022103LC01-E1_2**	132.07	51.95	2.542
**Acerola EC 2022103LC01-E2_2**	143.58	56.74	2.531
**Acerola EC 2022103LC02-E1_2**	136.13	54.26	2.509
**Acerola EC 20303-1 E1_2**	117.65	72.66	1.619
**Acerola EC 20303-1 E2_2**	121.81	75.28	1.618
**Acerola EC 20303-2 E1_2**	101.32	62.72	1.615
**Acerola EC 20303-2 E2_2**	104.45	65.09	1.605
**Acerola EC 20315-1-E1_2**	146.95	86.61	1.697
**Acerola EC 20315-1-E2_2**	131.09	78.35	1.673
**Acerola EC 20316-1-E1_2**	113.92	64.70	1.761
**Acerola EC 20364-1-E1_2**	106.63	55.90	1.908
**Acerola EC 20364-1-E2_2**	113.11	58.84	1.922
**Acerola EC 20364-2-E1_2**	94.18	48.77	1.931
**Acerola EC 20364-2-E2_2**	114.37	59.24	1.931
**Acerola EC B0296600-E1_2**	103.82	53.25	1.950
**Acerola EC B0296600-E2_2**	126.36	64.84	1.949
**Acerola EC B1186687-E1_2**	103.64	46.35	2.236
**Acerola EC B1186687-E2_2**	120.47	53.79	2.240
**Acerola EC F0296602-E1_2**	128.59	57.19	2.248
**Acerola EC F0296602-E2_2**	135.06	60.29	2.240
**Acerola EC H0296602-E1_2**	103.97	45.45	2.287
**Acerola EC H0296602-E2_2**	113.13	49.25	2.297
**Acerola EC J0296604-E1_2**	123.37	50.94	2.422
**Acerola EC J0296604-E2_2**	100.18	41.55	2.411
**Acerola EC K0296605-E1_2**	100.29	49.43	2.029
**Acerola EC K0296605-E2_2**	109.36	53.67	2.038
**Acerola EC L0296606-E1_2**	112.60	58.23	1.934
**Acerola EC L0296606-E2_2**	105.38	54.36	1.939

**Acerola EC L20310-1-E1_2**	108.52	67.10	1.617
**Acerola EC L20310-1-E2_2**	104.85	63.79	1.644
**Acerola EC L20315-2-E1_2**	92.18	63.10	1.461
**Acerola EC L20315-2-E2_2**	111.62	76.19	1.465
**Acerola EC L20316-1-E2_2**	109.41	62.50	1.751
**Acerola EC L20331-1-E1_2**	126.44	67.36	1.877
**Acerola EC L20331-1-E2_2**	119.95	63.20	1.898
**Acerola EC N0296607-E1_2**	112.20	56.65	1.981
**Acerola EC N0296607-E2_2**	120.74	60.81	1.986
**Acerola EC X0296699-E1_2**	110.62	58.62	1.887
**Acerola EC X0296699-E2_2**	120.10	63.30	1.897
**Acerola EC Z1156679-E1_2**	103.66	49.18	2.108
**Acerola EC Z1156679-E2_2**	104.27	49.37	2.112
**Acerola EC2021019LC01-E1_2**	83.35	51.78	1.610
**Acerola EC2021019LC01-E2_2**	113.57	70.58	1.609
**Acerola EC2021019LC02-E1_2**	91.02	52.76	1.725
**Acerola EC2021019LC02-E2_2**	95.33	55.18	1.728
**Acerola EC2021042LC02-E1_2**	98.48	60.78	1.620
**Acerola EC2021042LC02-E2_2**	102.59	62.89	1.631
**Mean**			1.995
**SD**			0.275
**RSD (%)**			13.80%

B4_8905: bucket centered at 4.89 ppm containing the doublet of ascorbic acid H1; B3_2595: bucket centered at 3.26 ppm containing the singlet of choline $(CH_3)_3$; NMR area in arbitrary unit.

Table S 2. Ingredients listed on product manufacturer labels

Food supplements (D.S.)	Composition	Galenic form
Acerola reference powder	Organic acerola extract powder titrated to 17% of Vitamin C	Powder
D.S.1	Organic acerola extract powder titrated to 17% Vitamin C	Powder
D.S.2	Acerola extract powder titrated to 15.44% of Vitamin C<br>Royal jelly<br>*Citrus paradisi* fruit and seed extract	Tablet
D.S.3	Acerola extract powder titrated to 16.99% Vitamin C<br>Maltodextrin	Tablet

	Cane Sugar<br>Dried rice syrup<br>Dextrose<br>Gum acacia<br>Anti-caking agents: magnesium carbonate. Talc<br>Natural flavor: berries<br>Sunflower oil	
D.S.4	Sucrose<br>Vitamin C<br>Dextrose<br>Anti-caking agents: calcium stearate. silicon dioxide<br>Acidifying agent: citric acid<br>Flavor<br>Acerola extract<br>Cynorrhodon extract<br>Sweet orange extract	Tablet
D.S.5	Sorbitol<br>Acerola extract powder titrated to 25% Vitamin C<br>Xylitol<br>Natural flavor: berries<br>Anti-caking agents: magnesium Stearate<br>Stevia	Tablet
D.S.6	Acerola extract powder titrated to 25% Vitamin C<br>Sorbitol<br>Xylitol<br>Cane Sugar<br>Anti-caking agents: magnesium stearate<br>Cherry flavor<br>*Citrus sinensis* bioflavonoids	Tablet
D.S.7	Acerola extract powder titrated to 17% Vitamin C<br>Vegetal cover	Capsule
D.S.8	Acerola extract powder with added Vitamin C<br>titrated to 17% Vitamin C<br>Blackcurrant powder<br>Cranberry powder<br>Anti-caking agents: calcium salts of fatty acids<br>Sodium saccharin	Tablet
D.S.9	Acerola extract powder<br>Sorbitol<br>Sucralose<br>Acesulfame potassium	Tablet

	Anti-caking agents: calcium salts of fatty acids<br>Naturel red fruit flavor<br>Anti-caking agents: calcium salts of fatty acids	
D.S.10	Sorbitol<br>Acerola extract powder<br>Anti-caking agents: calcium stearate<br>Vitamin C<br>Cherry flavor	Tablet

Table S3. Calculated (AA area/Choline area) ratio for ^{1}H-NMR spectra of 50 samples prepared from acerola powder

Sample code	Choline area B3_2490	AA area B4_8730	Ratio (AA area/ Choline area)
**acerola bio b1388901 c11_2**	20.14	52.31	2.597
**acerola bio c2118201 c11_2**	23.19	46.32	1.997
**acerola bio f1465301 c21_2**	21.69	45.10	2.080
**acerola bio f1465301 c31_2**	23.61	48.89	2.071
**acerola bio f1465301 c41_2**	22.00	45.65	2.075
**acerola bio f2118101 c41_2**	20.54	48.76	2.374
**acerola bio f2118202 c11_2**	23.26	42.82	1.841
**acerola bio h2096801 c21_2**	21.98	47.93	2.180
**acerola bio k1377601 c241_2**	20.70	46.50	2.247
**acerola bio k2011701 c101_2**	23.72	42.52	1.793
**acerola bio k2011701 c21_2**	23.82	42.75	1.794
**acerola bio k2011701 c61_2**	23.20	41.59	1.793
**acerola bio k2263301 c11_2**	20.26	44.79	2.211
**acerola bio l1388601 c41_2**	20.77	45.15	2.174
**acerola bio l1388601 c71_2**	20.40	43.49	2.132
**acerola bio n1185401 c51_2**	19.19	43.04	2.242
**acerola bio n1185401 c81_2**	18.32	41.46	2.263
**acerola bio n2253001 c21_2**	20.43	51.92	2.542
**acerola bio n2253001 c61_2**	19.59	50.13	2.559
**acerola Bio R0294501-C21_2**	19.09	39.78	2.083
**acerola Bio R0294501-C41_2**	19.36	28.88	1.491
**Acerola Bio R0294501-C61_2**	19.63	28.01	1.427
**acerola bio r2252901 c111_2**	20.14	46.56	2.312
**acerola bio r2252901 c151_2**	20.31	46.37	2.283
**acerola bio r2252901 c71_2**	19.68	45.27	2.300
**acerola bio t1185201 c111_2**	18.59	44.78	2.408
**acerola bio t1185201 c131_2**	18.34	44.18	2.409

**acerola bio t2142301 c41_2**	19.27	47.29	2.454
**acerola bio t2142301 c61_2**	19.10	47.06	2.464
**acerola bio t2142301 c81_2**	18.70	45.86	2.452
**acerola bio u1399301 c1_2**	20.57	45.87	2.230
**acerola bio u1399301 c11_2**	20.43	46.18	2.260
**acerola bio u1399301 c31_2**	20.26	45.95	2.268
**acerola bio u2020701 c101_2**	23.17	41.99	1.812
**acerola bio u2020701 c141_2**	23.21	42.69	1.840
**acerola bio u2020701 c21_2**	23.25	42.22	1.816
**acerola bio u2020701 c61_2**	22.87	41.42	1.811
**acerola bio w0262001 c101_2**	18.07	41.54	2.299
**acerola bio w0262001 c11_2**	17.83	40.87	2.292
**acerola bio w0262001 c41_2**	17.88	40.13	2.244
**acerola bio w0262001 c81_2**	18.19	41.30	2.270
**acerola bio w0262001-c121_2**	18.31	41.64	2.274
**acerola bio w0262001-c141_2**	18.43	41.92	2.275
**acerola bio w0262001-c161_2**	17.73	40.03	2.258
**acerola bio w0262001-c181_2**	18.29	40.44	2.211
**acerola bio w0262001-c211_2**	18.35	40.75	2.221
**acerola bio w0262001-c51_2**	17.72	40.19	2.268
**acerola bio x1422201 c111_2**	21.19	44.67	2.108
**acerola bio x1422201 c131_2**	22.00	46.19	2.099
**acerola bio x1422201 c231_2**	21.97	45.89	2.089
**Mean**			2.159
**SD**			0.254
**RSD (%)**			11.77%

B3_2490: bucket centered at 3.25 ppm containing the singlet of choline $(CH_3)_3$; B4_8730: bucket centered at 4.87 ppm containing the doublet of ascorbic acid H1; NMR area in arbitrary unit

Table S4. Repeatability of the qNMR method based on ^{1}H-NMR

**Experiments**	**AA area B4_8695**	**TSP area B0_0170**
Ascorbic Acid (Batch 1966)-1	133.26	25.34
Ascorbic Acid (Batch 1966)-2	132.63	25.13
Ascorbic Acid (Batch 1966)-3	132.08	25.19
Ascorbic Acid (Batch 1966)-4	131.44	24.99
Ascorbic Acid (Batch 1966)-5	132.48	25.01
Ascorbic Acid (Batch 1966)-6	131,38	24.94
Ascorbic Acid (Batch 1966)-7	132.43	25.03
Ascorbic Acid (Batch 1966)-8	131.79	24.76

Ascorbic Acid (Batch 1966)-9	132.32	25.05
Ascorbic Acid (Batch 1966)-10	131.15	24.85
**Mean (n=10)**	132.09	25.03
**SD**	0.656	0.166
**RSD (%)**	0.50%	0.66%

B4_8695: bucket centered at 4.87 ppm containing the doublet of ascorbic acid H1; B-0_0170: bucket centered at 0 ppm containing the singlet of TSP; NMR area in arbitrary unit

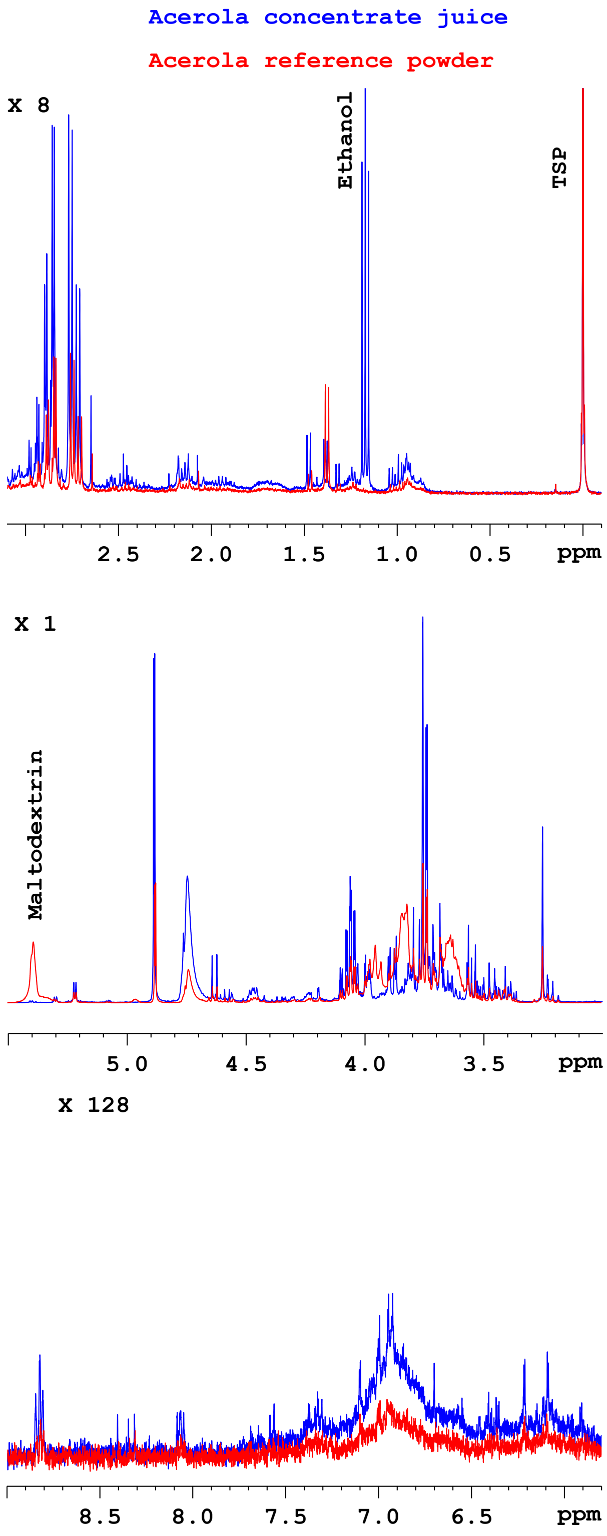

Figure S1. Overlay of 1D ^{1}H-NMR spectra of acerola concentrate juice (blue) and acerola reference powder (red) (400 MHz, 300 K, D_2O, pH 3.35– 20 mM TSP)

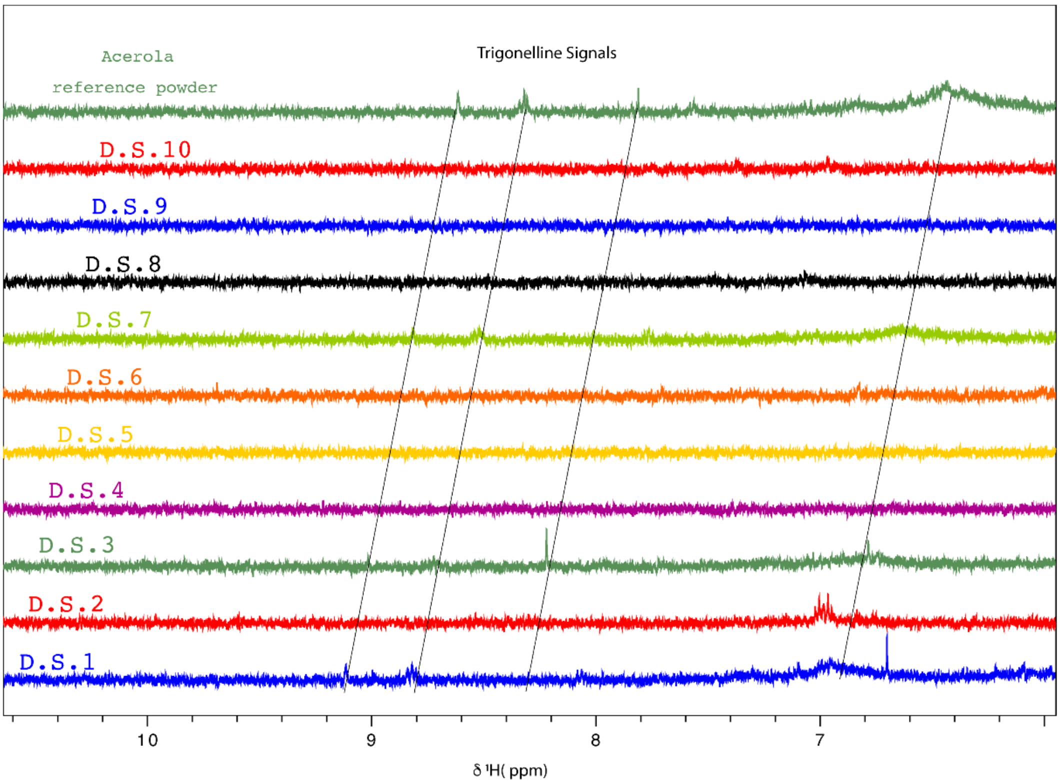

Figure S2. Zoom in on signals observed between 6 and 10 ppm (aromatic region) on food supplement ¹H-NMR spectra (400 MHz, 300 K. D_2O, pH 3.35, 20 mM TSP)

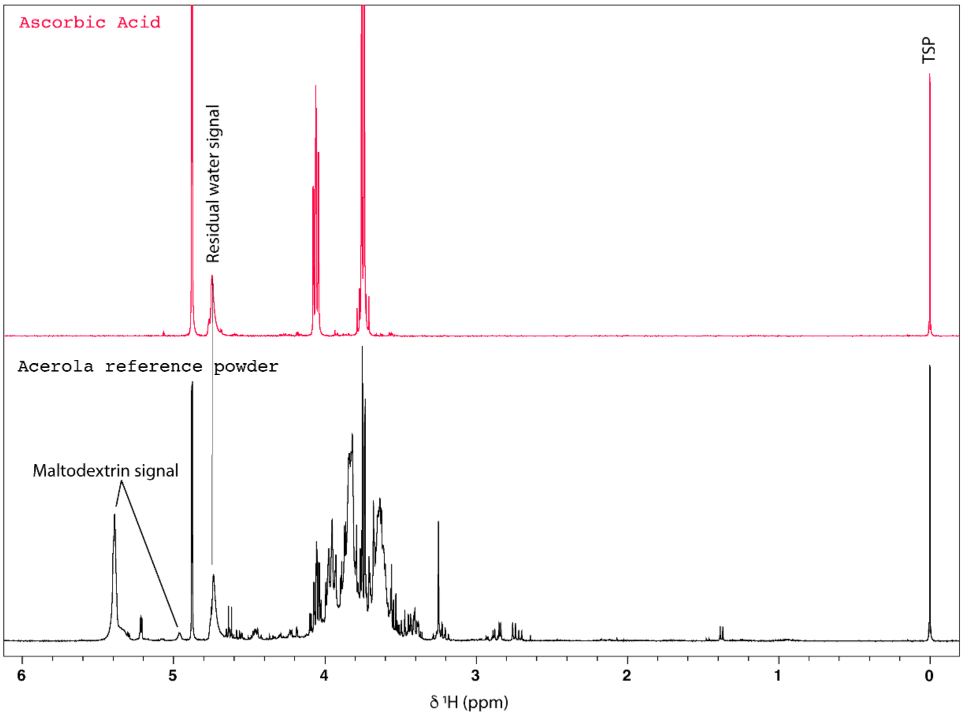

Figure S3. Comparison between spectra of ascorbic acid (red) and of the sample of solution prepared from acerola reference powder (black) (400MHz. 300 K. D_2O pH 3.35, 20 mM TSP)
